# What can we learn about immediate memory from the development of children's free recall?

**DOI:** 10.1080/17470218.2014.995110

**Published:** 2015-02-16

**Authors:** Christopher Jarrold, Debbora Hall, Caroline E. Harvey, Helen Tam, John N. Towse, Amy L. Zarandi

**Affiliations:** ^a^ School of Experimental Psychology, University of Bristol, Bristol, UK; ^b^ Psychology, Lancaster University, Lancaster, UK

**Keywords:** Primary memory, Free recall, Immediate memory development

## Abstract

We ask the question: Which aspects of immediate memory performance improve with age? In two studies, we reexamine the widely held view that primary memory capacity estimates derived from children's immediate free recall are age invariant. This was done by assessing children's immediate free-recall accuracy while also measuring the order in which they elected to recall items (Experiment 1) and by encouraging children to begin free recall with items from towards the end of the presented list (Experiment 2). Across samples aged between 5 and 8 years we replicated the previously reported age-related changes in free-recall serial position functions when aggregated across all trials of the standard task, including an absence of age differences in the recency portion of this curve. However, we also show that this does not reflect the fact that primary memory capacity is constant across age. Instead, when we incorporate order of report information, clear age differences are evident in the recall of list-final items that are output at the start of a participant's response. In addition, the total amount that individuals recalled varied little across different types of free-recall tasks. These findings have clear implications for the use of immediate free recall as a means of providing potential indices of primary memory capacity and in the study of the development of immediate memory.

The study of immediate memory has an extensive history in experimental psychology. For example, James (1890) drew a distinction between “primary” and “secondary” memory, with the former referring to the subset of information that is currently available to conscious access and that can be immediately recalled as a result, and the latter being information that must be retrieved from “memory proper”. Subsequent authors subsumed this distinction into models of short-term and working memory (e.g., Atkinson & Shiffrin, 1968; Baddeley & Hitch, 1974), and one can certainly draw a parallel between the notion of primary memory and the concept of short-term memory as assessed by measures of immediate serial recall or so-called “simple span” tasks. Indeed, current models of short-term memory often assume that the capacity of this system is determined by the number of items that one can keep active within some “focus of attention” or “region of direct access” (Cowan, 2001; Cowan et al., 2005; Oberauer, 2002), thereby drawing a direct link between short-term memory performance and the concept of primary memory as defined by James.

Having said this, the concepts of primary and secondary memory are not synonymous with short- and long-term memory, respectively, because they are less clearly tied to particular time-scales over which forgetting might occur. Rather, they are definitions that focus more on different underlying *processes*—the maintenance of information in a consciously accessible state in the case of primary memory, the retrieval of information by effortful and strategic search in the case of secondary memory—than on separable cognitive *structures*. In line with this, primary memory has recently become the focus of considerable research attention in areas that go beyond the study of immediate serial recall and the related concept of short-term memory. This renaissance is largely due to work by Unsworth et al. (e.g., Unsworth & Engle, 2006, 2007a, 2007b), who have argued that both primary and secondary memory underpin working memory performance as measured using “complex span” tasks that combine short-term storage with potentially distracting processing activity (see Conway et al., 2005). Performance on complex span tasks is known to be a strong predictor of individual differences on a range of cognitive traits, including measures of intelligence and academic attainment in both adults (e.g., Kane, Hambrick, & Conway, 2005; Oberauer, Schulze, Wilhelm, & Süß, 2005) and children (Bayliss, Jarrold, Gunn, & Baddeley, 2003; De Smedt et al., 2009; Nevo & Breznitz, 2011; Swanson, 2011). However, Unsworth et al. have suggested that, rather than reflecting the presence of a unitary underlying construct of working memory, this predictive power of complex span tasks is better understood in terms of the independent contributions of primary and secondary memory capacity to task performance (Unsworth, Spillers, & Brewer, 2010; see also Mogle, Lovett, Stawski, & Sliwinski, 2008; Shelton, Elliott, Matthews, Hill, & Gouvier, 2010).

In order to properly test this account without falling foul of problems of circularity, one clearly needs to derive estimates of primary and secondary memory capacity that are themselves independent of complex span performance. One potential means of extracting these estimates is to use a free-recall task, in which the participant is presented with a list of words and is then asked to recall as many of these words as they can in any order they wish. Waugh and Norman (1965) first suggested that free recall depends on both primary and secondary memory capacity. They argued that, provided sufficient items are presented in an immediate free-recall task, secondary memory capacity can be estimated from the level at which recall performance asymptotes on the middle serial positions of the list; although these items would have been held in primary memory at the point of their presentation, they would have been displaced to secondary memory by the subsequent presentation of later items on the list. In contrast they suggested that primary memory capacity can be gauged from the additional benefit to recall seen on list-final items (the final 7 list items in their analyses), which they assumed would still be available to conscious access. Tulving and Colotla (1970) further developed this method of estimating primary and secondary memory capacity on the assumption that participants begin immediate free recall by first outputting the list-final items that are currently in primary memory, before then performing a search of secondary memory in an attempt to recall earlier list items (see also Tulving, 1968). They therefore divided items into those recalled from primary and secondary memory using the intratrial retention interval (ITRI)—that is, the number of other items that were either presented or recalled between the presentation and recall of the given item. They argued that any item with an ITRI of 7 or less should be classified as having been recalled from primary memory.

It is important to note, however, that many other theoretical accounts would argue against the view that free recall depends on the functioning of two distinct systems and would favour a more unitary explanation of performance (Brown, Neath, & Chater, 2007; Howard & Kahana, 2002).^1^

^1^Farrell (2012) has proposed an account of free recall that, potentially, provides something of a reconciliation of these views. Although essentially a unitary account, based on the common hierarchical structuring of memory into discrete episodic clusters (see General Discussion for a more thorough treatment), Farrell's (2012) model assumes that individuals can recall just-presented information from the currently open episodic cluster without the need to first access it. This account therefore shares features with models that assume that just-presented items have greater accessibility than all other material by virtue of being active in some form of focus of attention (e.g., Cowan, 2001; Jonides et al., 2008; McElree, 2006; see also Nee & Jonides, 2011; Öztekin, Davachi, & McElree, 2010). Nevertheless, a number of recent studies have employed free-recall tasks with the expressed purpose of extracting primary memory estimates from them (De Alwis, Myerson, Hershey, & Hale, 2009; Gibson, Gondoli, Flies, Dobrzenski, & Unsworth, 2009; Gibson et al., 2013; Unsworth, Brewer, & Spillers, 2011; Unsworth et al., 2010). Consequently, one focus of the current paper was to ask whether the assumptions underlying the simple partitioning of free-recall performance into primary and secondary memory components are valid, and, in particular, whether free recall provides the direct index of primary memory capacity in children that one would assume given the recent interest in this possibility in the adult literature (cf. Unsworth & Engle, 2007a).

Another reason for raising this question is that the one recent study that has attempted to extract primary and secondary memory capacity estimates from children's free recall came to what appear to be surprising conclusions. De Alwis et al. (2009) examined the development of primary and secondary memory in the context of free recall using the Words List test of the Children's Memory Scale (Cohen, 1997). In this task, participants are presented with a 14-item word list that they have to recall immediately, followed by further trials in which learning is assessed by reminding individuals of forgotten items. Recall of a novel distractor list and delayed recall of the original list are also assessed. This task was given to 57 children aged between 6 and 16 years, who, as a group, showed marked primacy and recency in their recall on the 1st trial of the target list and on the distractor list. However, De Alwis et al. did not record the order in which participants recalled list items and so were unable to conduct an analysis along the lines suggested by Tulving and Colotla (1970). Instead, for the purposes of their analyses they divided the presented list up into three subsections that corresponded to the first four, the middle six, and the final four items on the list, and they assumed that the last four items on the list would have been held in primary memory (cf. Moely, 1977). They then contrasted performance on these final items with recall of the initial subset, which they assumed was held in secondary memory. They found marked age effects on recall of the initial list items, but not on recall of the final list items. As a result, they suggested that “children's secondary memory improves with age, whereas their primary memory does not” (De Alwis et al., 2009, p. 929).

Although striking, this claim is not unprecedented and has been advanced in at least two previous studies of children's immediate free-recall performance (Cole, Frankel, & Sharp, 1971; Thurm & Glanzer, 1971; see Dempster & Rohwer, 1983; Jablonski, 1974). However, it remains a surprising one to make because there is considerable reason to believe that children's primary memory does in fact develop substantially. Given that primary memory is defined as the subset of information currently held active in mind (Unsworth & Engle, 2007a; Waugh & Norman, 1965), then an individual's ability to recall a just-presented list in correct serial order should provide a reasonable index of its capacity (see Unsworth & Engle, 2007b). As already noted, the concepts of primary memory and short-term memory are arguably synonymous with one another (Baddeley, 1986), and verbal span tasks, in which participants simply have to repeat a just-presented word or digit list, are accepted measures of verbal short-term memory and clearly show substantial developmental improvements across early and middle childhood (see Dempster, 1981; Gathercole, 1999). Furthermore, recent studies have shown a much greater commonality between adults’ free-recall and serial-recall performance than has previously been assumed (Bhatarah, Ward, Smith, & Hayes, 2009; Grenfell-Essam & Ward, 2012; Spurgeon, Ward, & Matthews, 2014; Ward, Tan, & Grenfell-Essam, 2010). Similarly, other studies that have used alternative methodologies to assess children's capacity to hold information active in mind in their “focus of attention” have also produced capacity estimates that show considerable developmental change (Cowan et al., 2005; Hall, Jarrold, Towse, & Zarandi, 2015).

This raises the possibility that De Alwis et al. (2009) were premature to assume that the final items on a free-recall list are necessarily held in primary memory. As the Tulving and Colotla (1970) procedure makes clear, this assumption depends on individuals beginning their recall with items towards the end of the just-presented list. If participants begin with initial list items, then their ability to recall the final items on the list would, instead, depend on secondary memory capacity under this dual-system account (Craik & Birtwistle, 1971). In addition, if older individuals are more likely to begin recall at the start than at the end of the list, then age differences in recall of these initial list positions are very likely to be observed (cf. Unsworth et al., 2011).

A crucial point is that earlier studies of children's immediate free recall that have led to the claim of development invariance in primary memory (Cole et al., 1971, Thurm & Glanzer, 1971) have also not reported the order of participants’ recall. This substantially constrains the conclusions that can be drawn from them regarding the development of either primary or secondary memory (cf. Cuvo, 1975). Although there is recent work that has estimated adolescents’ primary and secondary memory capacities from immediate free recall using the Tulving and Colotla (1970) method, and which has done so by rightly focusing on those individuals who tended to commence recall with list-final items (Gibson et al., 2009), that study did not examine developmental differences in these estimates. The main objective of the current pair of studies was to rectify this situation and to properly examine age-related changes in potential indices of primary memory capacity by recording output order information from immediate free recall (Experiment 1) and by encouraging individuals to begin free recall towards the end of the list (Experiment 2). As already noted, a subsidiary aim was to examine the extent to which one can extract a valid measure of primary memory capacity from children's free-recall performance.

## EXPERIMENT 1

The first experiment examined the development of immediate free recall across a sample of 5- to 6-year-olds and a sample of 7- to 8-year-olds. A standard immediate recall task was given to both age groups, using a broadly similar procedure to that employed by De Alwis et al. (2009). However, as well as recording individuals’ recall accuracy across the serial positions of each list, the order in which participants outputted each item during their recall was noted. Items were presented at the rate of 1 item per second. This relatively rapid presentation rate was adopted for two reasons. First, it was expected to discourage any strategic rehearsal processes that might otherwise take place during extended intervals between items, and that might also differ between age groups independently of any differences in primary memory capacity (e.g., Lehmann & Hasselhorn, 2007). Indeed in their study of the capacity of the focus of attention in children, Cowan et al. (2005) explicitly noted that “rapid or unpredictable” presentation is one method that can be used to reduce the contribution of strategic influences to the estimate of this capacity (or, in our terms, primary memory). Second, it allowed for a comparison with the De Alwis et al. (2009) study where the same presentation rate was employed. Having said this, while De Alwis et al. (2009) employed 14-item lists, the current study presented 9-item lists to both age groups, partially to reduce fatigue effects in this somewhat younger sample, but also because other work has shown that even adults tend to begin their free recall with list-final items once lists exceed five items in length (Ward et al., 2010). Indeed, Ward et al. (2010) found that adults commenced immediate free recall from 9-item lists with one of the final four items on the list on approximately 70% of occasions (and this figure was not substantially different for longer lists, including 14-item lists). Similarly, Thurm and Glanzer (1971) varied list length from 2 to 7 in their study of 5- and 6-year-olds’ free recall and showed standard, and consistent, serial position curves for list lengths 5 to 7. Consequently, though shorter than the list lengths used in many previous studies of children's free recall, a 9-item list length was assumed to be sufficiently long to produce expected serial position effects and to allow participants to elect begin their recall towards the end of the list.

### Method

#### Participants

Parental consent was obtained from a total of 136 pupils in Year 1 and Year 3 of two local primary schools providing mainstream education and with close to national average levels of pupil attainment. Four participants did not provide data due to absences or noncompliance. Consequently, in total, data from 70 Year 1 pupils (42 males) and 62 Year 3 pupils (23 males) were analysed. The mean age for Year 1 children was 6:03 (range = 5:09–6:08), and for Year 3 children was 8:03 (range = 7:09–8:09).

#### Materials

A corpus of 81 words was selected for use in the free-recall task. Care was taken to ensure that the words employed would be familiar to all participants. Words were therefore concrete nouns taken from the set found in Morrison, Chappell, and Ellis (1997) and were selected on the basis of age-of-acquisition ratings provided in that work. The “objective age-of-acquisition” rating provided by Morrison et al. (1997) corresponds to the age at which 75% of children are expected to know a given word. The mean value of this rating for the word set was 42.3 months (*SD* = 13.7), and the maximum value was 70.8 months, corresponding to the lowest age of the youngest children in the study (see above). Twenty-eight of the items in the pool contained two syllables, and the remaining 53 were one-syllable words. These words were presented both auditorily and pictorially. Where necessary, silence was added to the end of an utterance so that all audio recordings were 750 ms in duration. The pictorial representations of the items consisted of black-on-white line drawings, the majority of which were adapted from Snodgrass and Vanderwart (1980). Additional drawings for items not provided by the Snodgrass set were also created.

#### Procedure

Participants were tested in a single session lasting approximately 20 minutes that also contained a number of other memory tasks not reported here. The free-recall task contained eight test trials, preceded by a single practice trial, each of which consisted of the sequential presentation of a nine-word list. Stimuli were presented both visually and auditorily on a laptop computer at a rate of 1 item per second. This dual mode of presentation was employed because participants were too young to read written stimuli, and presenting pictures without an accompanying verbal label could lead to incorrect encoding if a participant generated a non-normative label for an image. Similarly, presenting stimuli in an auditory format only would lead to the risk of participants mishearing an item, particularly given the use of a relatively large and open set of stimuli. At the end of each trial, recall was prompted by the appearance of a cartoon character. Participants were instructed that they could recall the items in any order. Responses and their order were recorded by hand by the experimenter. To maximize the accuracy of this scoring, the experimenter utilized a response sheet that listed the items presented on each trial and simply annotated the order in which any item was recalled; any intrusions were also noted.

### Results

#### Analysis of recall performance aggregated across all trials regardless of output order

**Figure 1:**
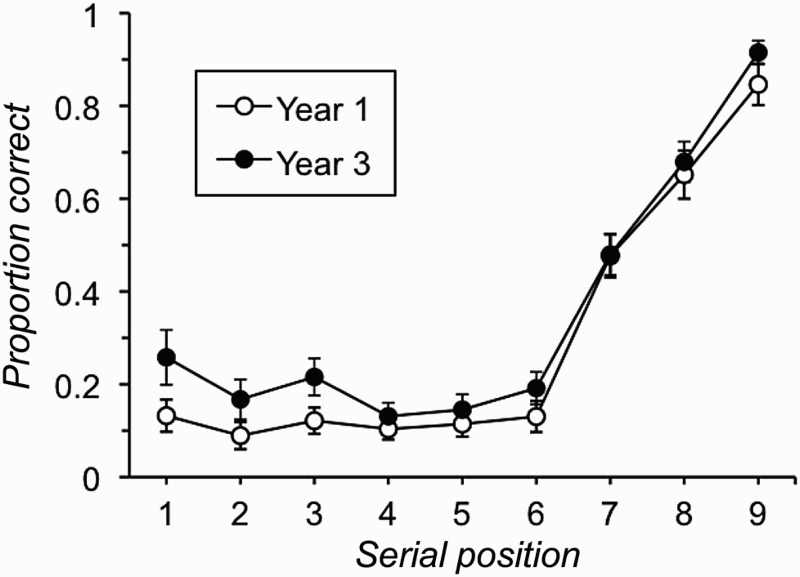
Free-recall accuracy by serial position and age group for all trials in Experiment 1 (error bars are 95% confidence intervals).

In order to compare the serial position effects in the current data with those reported by De Alwis et al. (2009), an initial analysis focused on average recall by serial position across all trials of the task. These data are plotted for each age group in Figure 1. The graph indicates that all participants showed relatively little primacy in their free-recall performance, with the serial position curves being marked by poor recall for items 1 to 6 in the list and better performance on items 7 to 9 with a strong recency gradient across these latter positions. Another point to note is that the performance of the two age groups appears to be similar on the final three positions of the list, but differs more clearly across the first six list positions (cf. De Alwis et al., 2009). A formal analysis that compared the two age groups across the nine serial positions of the list revealed a significant main effect of age group, *F*(1, 130) = 32.62, *p* < .001, *MSE* = 0.10, 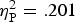, due to poorer overall recall of items in the Year 1 (*M* = 2.67, *SD* = 0.50) than in the Year 3 (*M* = 3.18, *SD* = 0.54) children. However, this was qualified by a significant interaction between age group and serial position, *F*(8, 1040) = 2.02, *p* = .042, *MSE* = 0.03, 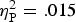. Given our interest in the claim for differential age effects on the different portions of the serial position curve, post hoc analysis of this interaction examined the simple main effect of age group on average recall across the first three, the middle three, and the final three serial positions separately. These showed that the effect of age group was significant on the first three, *F*(1, 130) = 21.23, *p* < .001, *MSE* = 0.02, 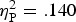, and the middle three items, *F*(1, 130) = 8.08, *p* = .005, *MSE* = 0.01, 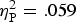, but not on the final three items on the list, *F*(1, 130) = 1.83, *p* = .179, *MSE* = 0.02, 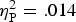.

#### Analyses of output order data

The above analysis therefore replicates De Alwis and colleagues’ (2009) findings in showing that age differences in children's free recall are apparent on the early-presented but not the late-presented items on the list. However, the fact that the order of participants’ responses was recorded in this experiment allows for two further analyses that are standardly employed in the adult literature (see Farrell, 2012; Howard & Kahana, 1999; Kahana, 1996; Ward et al., 2010), but have not been reported in previous studies of children's free recall. The first of these is an analysis of the lag between successive responses in an individual's output—that is, the difference between the positions in the input list of two successive responses in the output. This shows the extent to which participants recall in forwards serial order (recalling item X and then item X + 1 from the input list is a lag of +1) as well as all other possible movements between items. Typically, researchers analyse individuals’ “lag conditional response probabilities” or lag-CRPs (Kahana, 1996), and this approach was adopted here. This involves conditionalizing the likelihood of an individual making a response transition of a given lag against the opportunities for that transition to have taken place (e.g., an individual who always begins recall with the last item on the list can never make a +8 transition however many responses they go on to produce).

**Figure 2:**
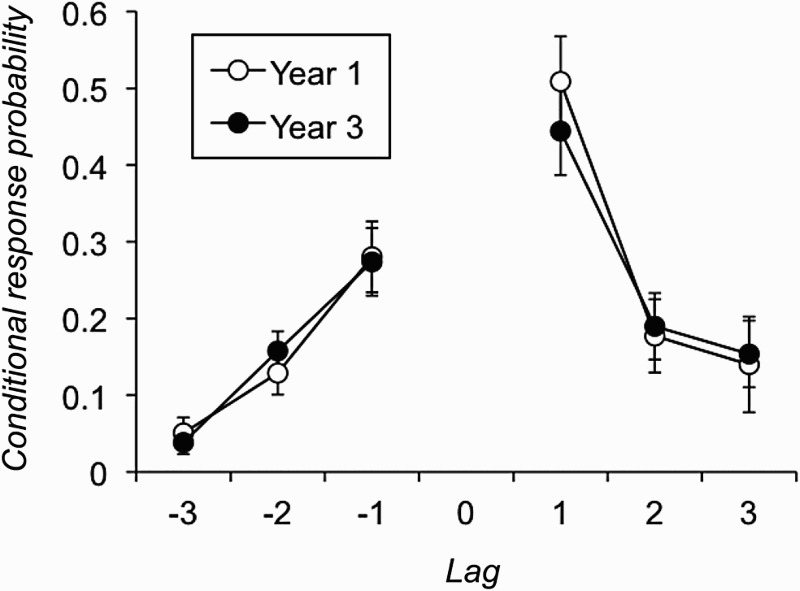
Lag conditional response probabilities in Experiment 1 (error bars are 95% confidence intervals).

Specifically, for each individual the number of −8 to +8 lag transitions made across all eight trials of the task were conditionalized against the opportunities to make such transitions. On 76 of the 1056 trials in the experiment a participant only recalled one item, thereby providing no lag-CRP data on that trial. However, 122 children produced lag-CRP data on all eight trials of the task, four produced data on seven trials, four on six, one on four, and one on just two trials. In addition, if there were no opportunities to make a certain lag transition across all trials then this cell was coded as missing data. As a result, not all individuals provided CRPs for all possible lags. In order to maintain an appropriate number of participants for analysis (cf. Kahana, 1996), we therefore considered the two age groups’ tendency to produce −3 to +3 lags, and these data are plotted in Figure 2. Sixty-one Year 1 and 55 Year 3 children provided data for this comparison.

Analysis of variance of these CRPs with the factors of lag (−3 to −1, +1 to +3) and age group revealed a significant main effect of lag, *F*(5, 570) = 83.275, *p* < .001, *MSE* = 0.03, 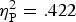; as Figure 2 shows, +1 lags were particularly common, a finding observed in the adult literature and termed the “lag recency effect” (Howard & Kahana, 1999). Neither the main effect of age group, *F*(1, 114) = 0.20, *p* = .654, *MSE* = 0.02, 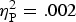, nor the interaction between lag and age group, *F*(5, 570) = 1.01, *p* = .414, *MSE* = 0.03, 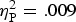, was significant. A further a priori analysis examined +1 lags only in the full sample of 132 participants and showed that the effect of age group on the tendency to run in forwards serial order was nonsignificant, *F*(1, 130) = 1.85, *p* = .176, *MSE* = 0.06, 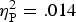.

**Figure 3:**
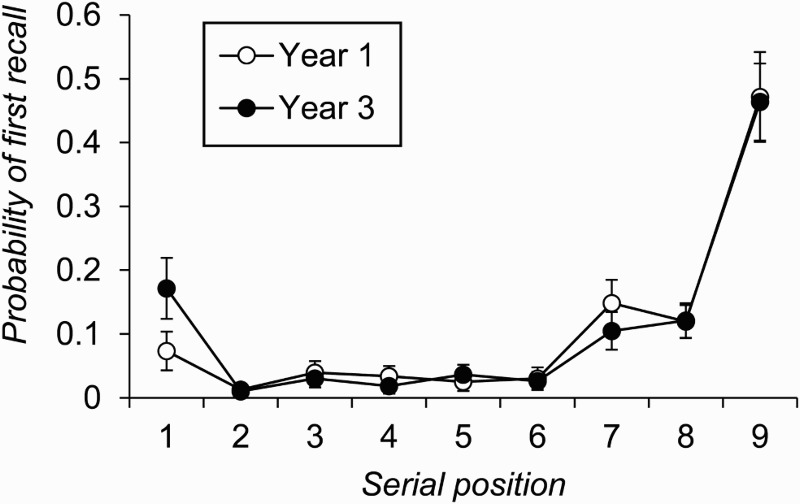
Probability of first recall by serial position and age group, averaged across all eight trials in Experiment 1 (error bars are 95% confidence intervals).

The second analysis that involved information about the order of an individual's recall examined participants’ probability of first recall functions (Howard & Kahana, 1999)—that is, individuals’ tendency to start their output with items at different positions in the input list. Figure 3 plots the probability of first recall data—that is, the probability with which individuals in each age group began their recall at a given serial position on a list, averaged across the eight trials of the free-recall task. Analysis of these data with the factors of age group and serial position revealed a significant interaction between the two factors, *F*(8, 1040) = 2.65, *p* = .007, *MSE* = 0.02, 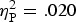. Post hoc analysis of the simple main effects of age group at each serial position showed a significant age difference only at serial position 1, *F*(1, 130) = 11.59, *p* = .001, *MSE* = 0.03, 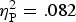, as individuals in Year 3 were more likely than those in Year 1 to begin recall with the first item on the list [the corresponding statistic for serial position 7 was *F*(1, 130) = 3.28, *p* = .072, *MSE* = 0.02, 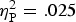].

#### Conditionalizing serial position functions by recall starting position

**Figure 4:**
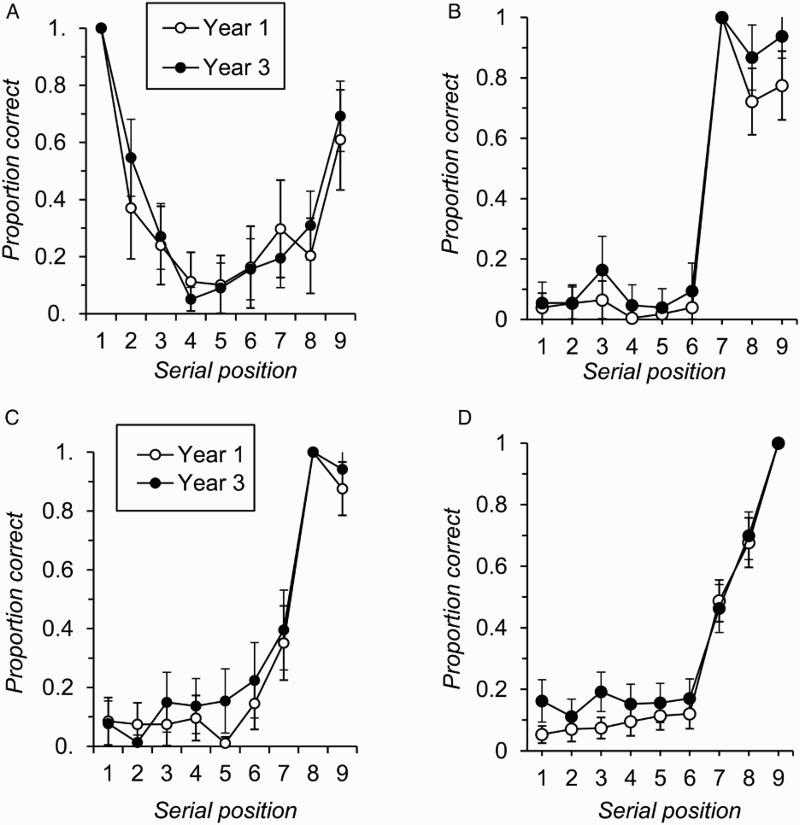
Free-recall accuracy by serial position and age group for trials where participants commenced recall with the item presented at list position 1 (Panel A), list position 7 (Panel B), list position 8 (Panel C), or list position 9 (Panel D) (error bars are 95% confidence intervals).

The final series of analyses integrated the previous two and was motivated by the fact that some individuals, particularly in Year 3, began recall with the first item on the just-presented list. This pattern is clearly problematic for the Tulving and Colotla (1970) method of estimating primary and secondary memory scores from free-recall performance. On a 9-item list of the kind employed here, outputting the first item first corresponds to an ITRI of 8, which would place that item in secondary memory under the typical adult scoring criterion. Given this problem, and the fact that the clear majority of trials involved first recall from position 1, 7, 8 or 9 of the list (81.3% of trials in Year 1, 86.1% in Year 3, see Figure 3), the effect of individuals commencing free recall at different list positions was examined by replotting serial position functions separately for trials when recall began at each of these four positions. These graphs are shown in Figure 4, and Table 1 summarizes the number of individuals in each age group who provided data for these figures and the average number of trials on which recall commenced at each position for each individual.

**Table 1.** Summary data for participants in each age group who began free recall at a particular list position in Experiment 1

	Starting position							
	1		7		8		9	
Year group	*n*	No. of trials	*n*	No. of trials	*n*	No. of trials	*n*	No. of trials
Year 1	23	1.78 (0.85)	47	1.79 (1.04)	47	1.44 (0.62)	63	4.21 (1.98)
Year 3	39	2.11 (1.52)	32	1.61 (0.83)	39	1.53 (0.71)	59	3.98 (1.96)

*Note:* Number of individuals starting at each position and the average number of trials started at that position by each individual are shown. Standard deviations in parentheses.

Table 1 clearly shows that not all individuals contributed data to each of the panels in Figure 4 (only 13 individuals across both age groups provided data for all four figures simultaneously). Consequently, analysis of these serial position curves was carried out for each starting position separately, and the interpretation of the patterns observed should bear in mind the fact that different participants contribute to each dataset. These analyses broke serial position down into start, middle, and final list position groups. Items 1 to 3 were classed as start positions, items 4 to 6 were termed middle positions, and the final positions were items 7 to 9. However, in each analysis the start position was excluded from the calculation of average recall in each triad because conditionalizing recall by start position necessarily produced ceiling performance at this position.

When recall began at serial position 1 (Figure 4, Panel A), the two groups showed broadly comparable levels of overall recall, *F*(1, 60) = 0.91, *p* = .344, *MSE* = 0.72, 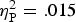, and the interaction between age group and serial position (triad) was not significant, *F*(2, 120) = 0.75, *p* = .471, *MSE* = 0.40, 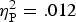. In contrast, when recall commenced with the item at position 7 (Figure 4, panel B), Year 3 children showed significantly greater recall accuracy, *F*(1, 77) = 10.58, *p* = .002, *MSE* = 0.32, 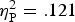. The interaction between factors was not significant at the 5% level, *F*(2, 154) = 2.38, *p* = .096, *MSE* = 0.35, 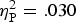, but was explored by further post hoc analysis of simple main effects. This showed that the two age groups did not differ significantly in performance on the start positions, *F*(1, 77) = 1.62, *p* = .208, *MSE* = 0.02, 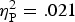, but that there was a trend towards a recall advantage for Year 3 children on middle positions, *F*(1, 77) = 3.82, *p* = .054, *MSE* = 0.01, 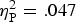, and a significant group difference in favour of Year 3 individuals on the final positions (positions 8 and 9), *F*(1, 77) = 5.82, *p* = .018, *MSE* = 0.08, 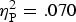.

When recall began with the item at serial position 8 (Figure 4, Panel C), Year 3 individuals again showed significantly greater recall accuracy than Year 1 children, *F*(1, 84) = 4.54, *p* = .036, *MSE* = 0.03, 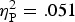, but there was no evidence of a reliable interaction with serial position, *F*(2, 168) = 0.96, *p* = .384, *MSE* = 0.35, 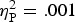. The main effect of age group was significant when recall commenced with the item from serial position 9 (Figure 4, panel D), *F*(1, 120) = 10.76, *p* = .001, *MSE* = 0.16, 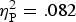. The interaction between factors was not significant at the 5% level, *F*(2, 240) = 2.60, *p* = .076, *MSE* = 0.21, 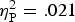. Supplementary post hoc analysis of simple main effects showed that Year 3 children significantly outperformed Year 1 individuals on the start positions, *F*(1, 120) = 11.90, *p* = .001, *MSE* = 0.18, 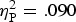, and middle positions, *F*(1, 120) = 4.31, *p* = .040, *MSE* = 0.16, 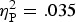, but not end positions (items 7 and 8), *F*(1, 120) < 0.01, *p* = .979, *MSE* = 0.25,   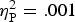.

### Discussion

The main aim of this first experiment was to examine the development of immediate free-recall performance and the extent to which this can be used to provide an index of children's primary memory capacity. Thus we tested the claim, made consistently in previous studies of children's immediate free recall, that primary memory does not develop in middle childhood (De Alwis et al., 2009; Jablonski, 1974; Thurm & Glanzer, 1971). The first point to note is that we essentially replicated earlier developmental studies of immediate free recall. The serial position functions that are shown in Figure 1 show less evidence of a primacy effect than those reported by De Alwis et al. (2009) and than is seen in adults’ immediate free-recall data (Farrell, 2012; Ward et al., 2010). However, De Alwis et al.’s sample included children aged up to 16 years, who might well be expected to show an adult pattern of performance, and primacy effects in immediate free recall are often absent in studies of younger children (Hasher & Clifton, 1974; Jablonski, 1974). It should also be noted that the use of auditory support alongside visual presentation of the memoranda in the current experiment is likely to increase the size of any recency effects on recall relative to those observed with visual-only presentation (Crowder & Morton, 1969). However, given that we adopted a fast presentation rate to discourage rehearsal of information, we see no strong reason to suspect that this would differentially have affected either age group's recall of list-final items. Indeed, when the nine presented items were split into three groups of three for analysis, Year 3 individuals recalled significantly more initial list items than Year 1 children but the groups did not differ reliably in their ability to recall final list items. This pattern is entirely consistent with the findings from previous studies of children's immediate free recall (Cole et al., 1971; De Alwis et al., 2009; Thurm & Glanzer, 1971) that, crucially, led these authors to conclude that primary memory does not develop with age.

Another finding that might, at least initially, be seen to be consistent with this claim is the fact that the conditional response probabilities produced by the two age groups were similar to one another (see Figure 2). In particular, both age groups showed the same tendency to recall items in forwards serial order. Serial ordering is a hallmark of short-term memory, and one might argue that the comparable tendency of the two age groups to make +1 lags in their responses shows comparable primary memory capacity. We return to this point below, but first note that while these aspects of our data appear to support the view that primary memory does not develop with age, other results favour the opposite conclusion.

In this regard, it is important to note that, while participants did show a strong tendency to begin recall with the last item on the list, there was a reasonable proportion of trials on which they began with the first list item. In addition, any tendency to begin recall at the start of the list was more common in Year 3 than in Year 1 children. Clearly, participants are likely to begin recall with items that they are confident of recalling correctly. Consequently, one would expect Year 3 children, who showed a greater tendency to start recall at the start of the list, to also show superior recall of initial list items (cf. Bjork & Whitten, 1974; Unsworth et al., 2011). By extension, it is possible that the age effects on recall of initial list items seen in previous studies (see above) reflect the fact that older children were more likely than younger children to begin recall at the start of the list.

It is, of course, possible that individuals selectively rehearse only initial list items to keep them active in primary memory, and that this is the reason why they begin recall with these items. However, without any corroborating evidence that this is occurring, one cannot be certain that initial list items are being recalled from this system. As a result, trials on which recall begins with items from the start of the list do not provide interpretable data for the measurement of primary memory capacity. Instead, according to Tulving and Colotla (1970), the assessment of primary memory capacity should focus solely on those trials when list-final items are recalled first. A point to note from Figure 3 is that while children were most likely to begin recall with the final item on the list, there was also a relatively strong tendency among all individuals to start recall with the 7th item. Indeed, participants were about as likely to begin recall with item 7 as they were with item 8.

Such a pattern of nonmonotonic decline in the probability of first recall from the end of the list is sometimes observed in adult data (Farrell, 2010; Howard & Kahana, 1999; Murdock, 1962; Murdock & Okada, 1970; Roberts, 1972) and is potentially in line with the claim that participants do, at least on some trials, begin recall by outputting the contents of primary memory. Assuming that primary memory is subject to something akin to a primacy gradient that leads to forwards serial recall (Farrell & Lewandowsky, 2002; Page & Norris, 1998), then a sensible approach to recalling from primary memory on an immediate free-recall task would be to begin recall X items in from the end of the list, where X corresponds to the number of items in primary memory that can be successfully recalled in forwards serial order (Howard & Kahana, 1999).^2^

^2^A potentially alternative explanation of the relative peak in the probability of first recall function at list position 7 is that participants might be grouping the nine-item list into three subgroups each of three items. One slight problem for this view is that there is no evidence in Figure 2 of any increase in probability of first recall for item 4, which would be the first item in the middle group under such a strategy. In addition, grouping the list in threes might simply be the consequence of participants having a primary memory capacity of about three items that allows them to recall from position 7 with relatively good accuracy (cf. Farrell, 2012).


If this relative peak in the probability of first recall functions shown in Figure 3 does indeed reflect participants outputting from primary memory, then the data most relevant to the question of whether primary memory indices from free recall change with age are the serial position functions that result when recall starts from position 7 (Figure 4, Panel B). In contrast to what is observed when performance is averaged across the whole experiment, on these trials Year 3 children recalled significantly more items than did Year 1 individuals from positions 8 and 9 of the list. If it is the case that initial free recall of list-final items reflects an individual's primary memory capacity, then these data suggest that primary memory capacity does increase with age in children. Although the lag-CRP analysis showed that the two groups had a comparable tendency to recall in forwards serial order, it is perfectly possible that this simply reflects the use of primary memory to drive response output. The same probability of forwards ordering for any given pair of successive responses might therefore be expected, even if the two groups did have different primary memory capacities.

## EXPERIMENT 2

The above discussion highlights the difficulty in making direct comparisons between the various panels shown in Figure 4, which are drawn from different subsets of participants in each year group in each case. Experiment 2 therefore borrowed a methodology from Dalezman (1976; see also Bruce & Papay, 1970; Cowan, Saults, Elliott, & Moreno, 2002) to prompt participants to begin their recall at a given position of the input list. Specifically, nine-item lists were again presented, but were divided into two sublists by a change in the presentation context. Participants were told in advance to prioritize recall of the second set of items. They were not explicitly instructed to commence recall with the very first of the second set items. However, we assumed that this manipulation would substantially increase the number of trials on which participants did in fact begin recall with the first item within the second set.

The number of items in this second set was varied from 2 to 4, in order to prompt recall from the 8th, 7th, or 6th position of the input list. We restricted the experimental manipulation to probe just these three positions, partly to avoid fatigue effects among our developmental sample, but primarily because of our focus on the development of potential indices of primary memory. Experiment 1 highlighted the relevance of data from trials in which recall commenced from position 7 of a nine-item list. However, it was less clear whether age effects were seen on list-final items when recall began at position 8; although age differences in total recall were seen in this case, they were driven by differences in recall of items at the start of the list (see Figure 4, Panel C). In addition, prompting recall from list position 6 allowed us to investigate whether participants of this age had recall capacities that exceeded three items. Recall from list position 1 was not prompted because including trials where recall was prompted from the first list position would have led participants to focus their attention, to some extent at least, on the start of the list. Rather, in this second experiment we wished to examine age differences in children's ability to recall list-final items under conditions in which they attempted to keep these items, and these items only, active in memory.

### Method

#### Participants

Parental consent was obtained from a total of 99 pupils in Years 1 and 3 of two local primary schools providing mainstream education, and with close to national average published attainment levels. Two participants did not provide complete data due to absences or noncompliance, but full data were obtained from 61 Year 1 pupils (29 males) and 36 Year 3 pupils (20 males). The mean age of the Year 1 group was 6:05 (range = 5:11–6:10), and the mean age of the Year 3 group was 8:05 (range = 7:10–8:10), in both cases closely matching the distribution of ages of the participants seen in Experiment 1.

#### Materials

Stimuli were drawn from a pool of 315 concrete nouns that were selected using the same criteria as those in Experiment 1. As in that experiment, approximately one third of items were two-syllable words, and two thirds were one-syllable items. Stimuli were again presented both auditorily and pictorially.

#### Procedure

Participants were tested in a single session lasting approximately 20 min. The task consisted of 30 test trials that were preceded by three practice trials. On any trial, the participant was presented with nine stimuli. These were presented sequentially at a rate of one item per second, with a pictorial representation of each to-be-remembered word, accompanied by simultaneous auditory presentation of that word. The first items on a trial were presented within a blue square in the centre of the screen, and the final items on a trial were presented within a red flash. Participants were told that they should try to recall both “blue” and “red” items, but that they would score more points for recalling red items. Three points were awarded to the participant for the recall of each red item, and 1 point was awarded for the recall of each blue item. Participants were shown the number of points that they had scored at the halfway point and at the end of the experiment.

There were three experimental conditions formed by variation of the number of blue and red items on a trial. In 5–4 trials, the first 5 list items were presented within the blue square, and the final 4 items were presented within the red flash; in 6–3 trials, the first 6 items were blue items, and the last 3 were red items; in 7–2 trials, the first 7 items were blue items, and the final 2 were red items. There were 10 trials in each condition, and trials from each condition were interleaved with one another in a predetermined, but apparently random, order.

At the end of any trial, recall of the red items was prompted by the appearance of a red flash. Participants were allowed 8 s to recall as many red items as they could, and these, and any erroneous responses, were recorded by the experimenter in the manner employed in Experiment 1. After 8 seconds had elapsed, a blue square appeared on the screen to prompt recall of the items presented in the blue phase of the trial. Participants had an unlimited time to recall blue items, and the experimenter recorded their responses and terminated the trial when they were satisfied that no more items would be recalled. Recall of red phase items always took place before the recall of blue phase items. Because of our interest in the recall of list-final items at the start of an individual's recall phase, the analyses reported below concentrate primarily on red phase recall of red items. However, we begin by reporting data that also include details of recall of blue items, in order to provide a summary of participants’ performance across the whole task.

### Results

#### Preliminary error analysis

An initial analysis examined task compliance in the two age groups in order to assess whether the procedure was properly understood, even by the younger participants. Across all trials, there was no significant effect of age group on the average number of blue items incorrectly recalled during the red recall phase per trial, *F*(1, 95) = 1.09, *p* = .299, *MSE* = 0.042, 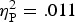, and the average number of such errors was low (Year 1, *M* = 0.21, *SD* = 0.21; Year 3, *M* = 0.17, *SD* = 0.20). Similarly, the effect of age group on the average number of red items recalled in the blue recall phase was nonsignificant, *F*(1, 95) = 0.10, *p* = .756, *MSE* = 0.007, 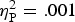, and this error pattern was even less common (Year 1, *M* = 0.08, *SD* = 0.08; Year 3, *M* = 0.08, *SD* = 0.09).

#### Analysis of recall performance aggregated across all trials regardless of output order

**Figure 5:**
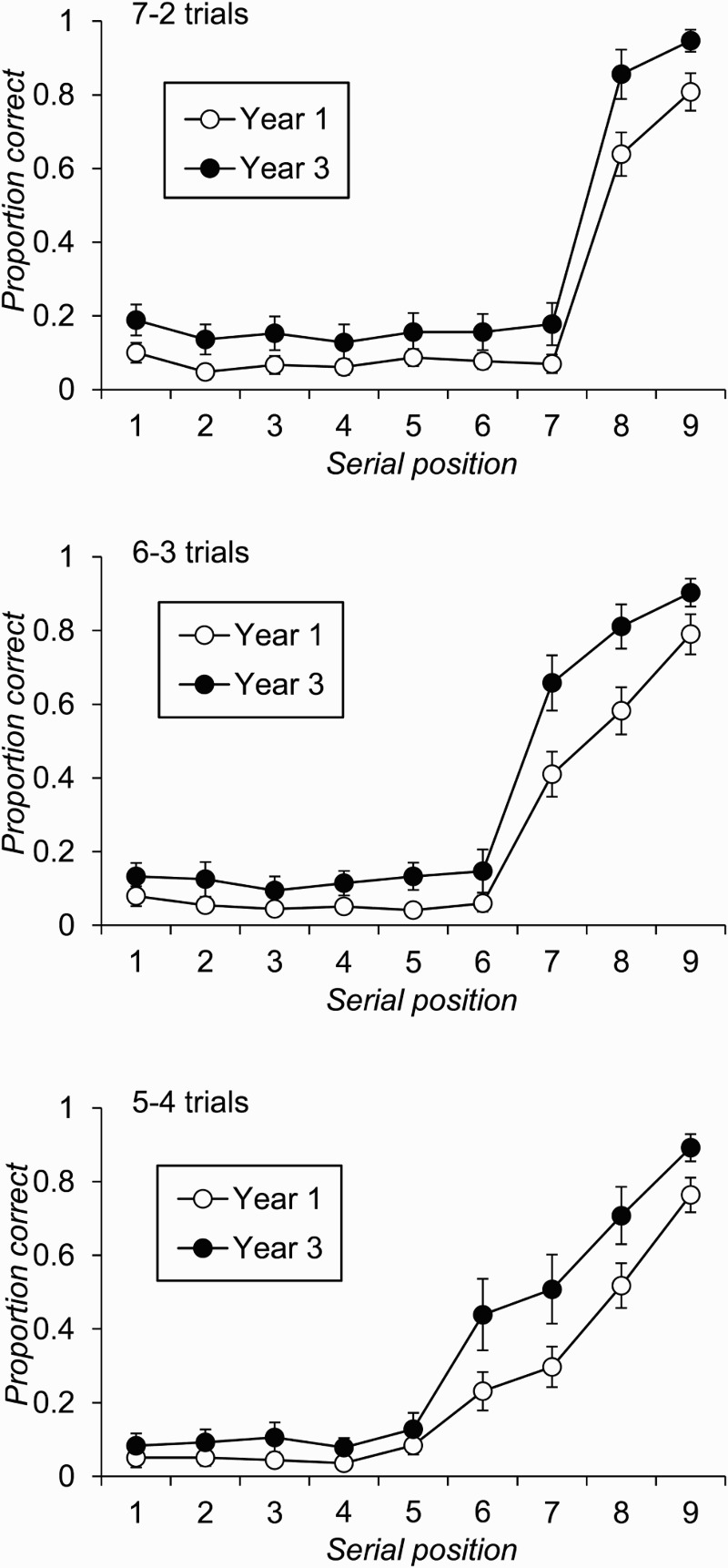
Free-recall accuracy by serial position and age group for red phase recall of red items and blue phase recall of blue items in Experiment 2 (error bars are 95% confidence intervals).

Average correct recall of each item in each recall phase is plotted for each age group in Figure 5. These graphs show strong recency effects in the correct recall of red list items and relatively flat serial position functions for correct recall of blue items. Analysis of these data was conducted, separately for each stimulus type (red or blue items), by averaging across serial position and comparing across conditions and age groups (an analysis of serial position effects for red phase recall is reported in a subsequent section of the results). The analysis of proportional correct recall of red items showed a significant effect of age group, *F*(1, 95) = 28.79, *p* < .001, *MSE* = 0.08, 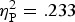, due to superior recall in Year 3 individuals. The effect of condition was significant, *F*(2, 190) = 205.43, *p* < .001, *MSE* = 0.01, 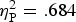, but the interaction between factors was not, *F*(2, 190) = 0.27, *p* = .765, *MSE* = 0.01, 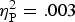. Post hoc analysis of the condition effect showed that proportional recall of red items was significantly higher in the 7–2 condition (*M* = .79, *SD* = .20) than in the 6–3 condition (*M* = .67, *SD* = .21), which in turn produced significantly greater proportional recall than the 5–4 condition (*M* = .52, *SD* = .19), *t*(96) = 9.63, *p* < .001, *t*(96) = 12.90, *p* < .001, respectively.

The corresponding analysis of correct recall of blue items in the blue recall phase similarly showed a significant main effect of year group, *F*(1, 95) = 26.57, *p* < .001, *MSE* = 0.01, 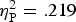. The effect of condition was significant, *F*(2, 190) = 20.97, *p* < .001, *MSE* = 0.02, 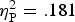, as was, in this case, the interaction between factors, *F*(2, 190) = 5.24, *p* = .006, *MSE* = 0.01, 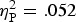. The interaction reflected a larger effect of condition among Year 3 individuals, *F*(2, 70) = 9.67, *p* < .001, *MSE* = 0.03, 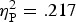, than among Year 1 participants, *F*(2, 120) = 8.99, *p* < .001, *MSE* = 0.01, 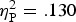. Conversely, the effect of year group was large and comparable in size for the 7–2 condition, *F*(1, 95) = 26.44, *p* < .001, *MSE* = 0.01, 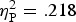, and the 6–3 condition, *F*(1, 95) = 26.13, *p* < .001, *MSE* = 0.00, 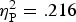, but smaller though still significant for the 5–4 condition, *F*(1, 95) = 10.28, *p* = .002, *MSE* < 0.01, 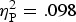.

The above analysis indicates that Year 3 children showed greater *proportional* recall of blue items than did Year 1 individuals, but particularly so on trials when they were required to recall only relatively few red items in the initial recall phase. One possible reading of these data is that Year 3 participants were able to hold a greater *total number* of items (red and blue) in mind than Year 1 individuals, so that provided the number of red items to recall did not exceed this total, they were able to also recall more blue items than their younger counterparts. To explore this possibility, we conducted an analysis of the total number of items correctly recalled across both phases of each trial of each condition. This revealed a significant main effect of age group, *F*(1, 95) = 38.34, *p* < .001, *MSE* = 1.66, 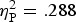, due to poorer total recall among Year 1 (*M* = 2.05, *SD* = 0.69) than Year 3 (*M* = 3.02, *SD* = 0.82) children. The main effect of condition was significant, *F*(2, 190) = 6.77, *p* = .001, *MSE* = 0.12, 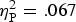, but the interaction between condition and year group was not reliable, *F*(2, 190) = 0.25, *p* = .776, *MSE* = 0.12, 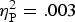. The condition effect reflected fewer total items being recalled in the 7–2 condition (*M* = 2.31, *SD* = 0.86) than in either the 6–3 (*M* = 2.48, *SD* = 0.93) or the 5–4 condition (*M* = 2.43, *SD* = 0.97). Indeed, there was no significant difference in total number of items recalled across the latter two conditions, *F*(1, 96) = 1.36, *p* = .247, *MSE* = 0.10, 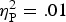.

#### Analyses of output order data

Following the analysis strategy of Experiment 1, output order data from participants’ successful recall of red phase items were first examined in terms of transitional conditional response probabilities, and then by examining probability of first recall data. Rather than considering CRPs at the full range of different lags, which varied across conditions, the lag-CRP analysis focused on children's tendency to recall in forwards serial order. Consequently, only +1 lags were subjected to analysis, which maximized the number of participants who provided data for this comparison. The +1 CRP is necessarily 1 for each participant when considering red phase recall in the 7–2 condition, as recalling the 9th item on the list is the only possibility for individuals’ second response if they begin recall with the 8th item on the list. In a similar vein, although a CRP analysis conditionalizes a given transition against the opportunity to make such a response, one might argue that random responding would necessarily lead to greater +1 lag transitions in the 6–3 than in the 5–4 condition simply because of the greater opportunity to make other transitions in the latter case. Given this, the analysis compared the +1 CRPs for Year 1 (*n* = 52) and Year 3 (*n* = 35) children for their red phase recall in the 6–3 and 5–4 conditions only, but with these probabilities averaged across these two conditions. The resultant analysis showed no significant effect of group on +1 CRPs, *F*(1, 85) = 1.05, *p* = .309, *MSE* = 0.02, 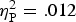 (Year 1, *M* = 0.84, *SD* = 0.16; Year 3, *M* = 0.87, *SD* = 0.11).

**Figure 6:**
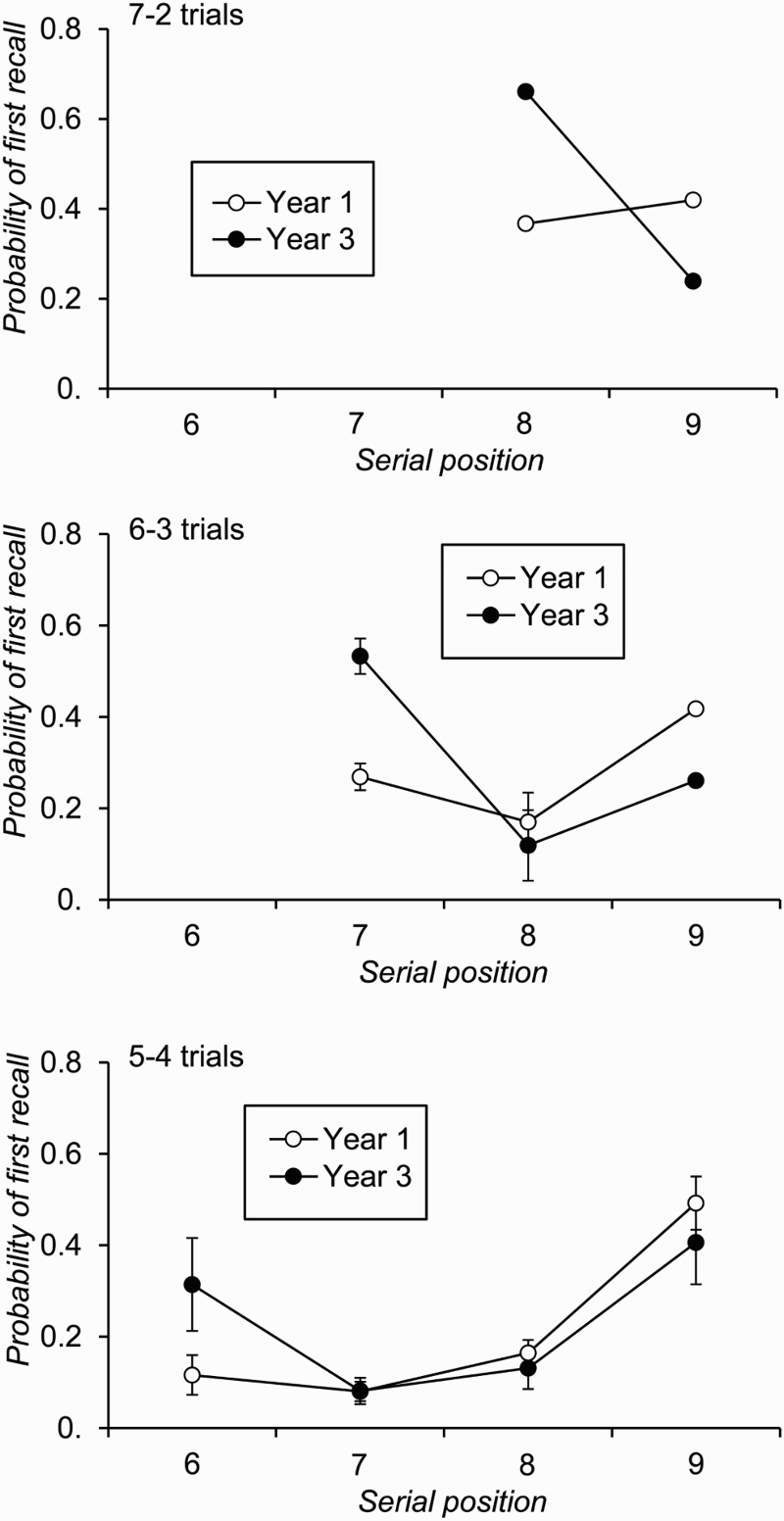
Probability of first recall by serial position and age group for the red recall phase of each condition of Experiment 2 (error bars are 95% confidence intervals).

Probability of first recall data are plotted by serial position in Figure 6. Because the main experimental aim of this second study was to encourage participants to begin their free recall from a certain point in the list, the analysis of these data was restricted to individuals’ likelihood of commencing recall with the first red item of the list (i.e., the 8th item in the 7–2 trials, the 7th item in the 6–3 trials, the 6th item in the 5–4 trials). Analysis of these data with the factors of condition and age group revealed significant main effects of condition, *F*(2, 190) = 91.69, *p* < .001, *MSE* = 0.02, 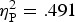, and of age group, *F*(1, 95) = 30.84, *p* < .001, *MSE* = 0.14, 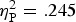. The interaction between factors was not significant at the 5% level, *F*(2, 190) = 2.46, *p* = .089, *MSE* = 0.02, 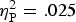. Post hoc analyses of simple main effects confirmed that Year 3 children were more likely than were Year 1 individuals to begin recall with the first red item in each condition, although the magnitude of this year group difference decreased somewhat with number of red items in the list: 7–2 condition, *F*(1, 95) = 34.02, *p* < .001, *MSE* = 0.06, 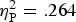; 6–3 condition, *F*(1, 95) = 21.71, *p* < .001, *MSE* = 0.07, 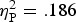; 5–4 condition, *F*(1, 95) = 16.24, *p* < .001, *MSE* = 0.05, 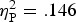.

#### Conditionalizing serial position functions by recall starting position

**Figure 7:**
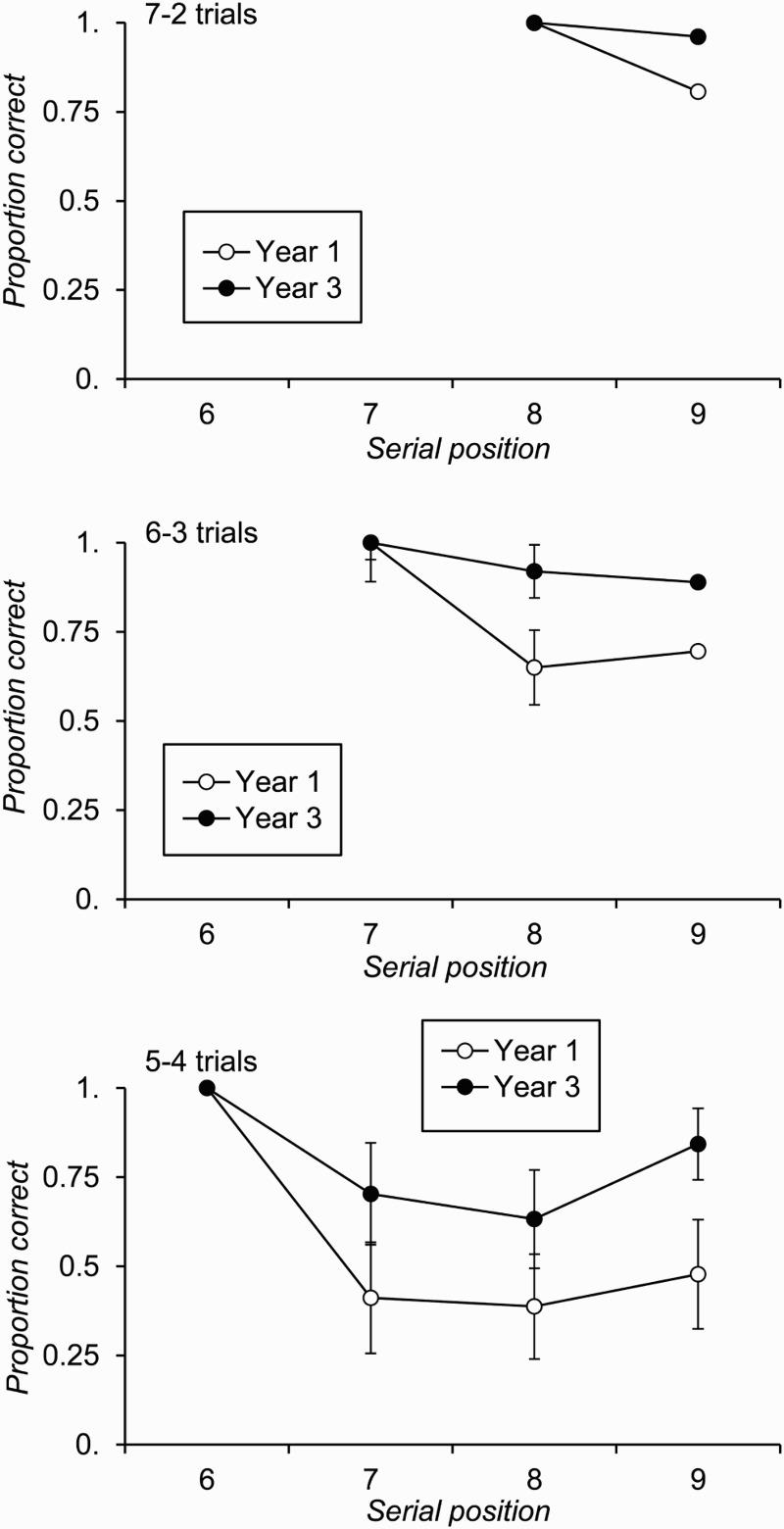
Free-recall accuracy by serial position and age group for trials where red phase recall commenced with the first red item in the list, plotted by condition of Experiment 2 (error bars are 95% confidence intervals).

As Figure 6 shows, children did not always begin recall in the red phase with the first red item on the list, although importantly this did occur on a sizeable proportion of trials, particularly in the Year 3 group. Therefore, to directly compare the results of this second experiment to those of Experiment 1 (cf. Figure 4), a final analysis of recall accuracy was conducted on data from only those trials on which individuals began red phase recall with the first red item in the just-presented list. Recall accuracy by serial position for such trials is plotted for each age group and condition in Figure 7. In the 7–2 condition, 57 Year 1 and 36 Year 3 children provided at least one trial's worth of data for this analysis. In the 6–3 condition, the corresponding numbers were 49 and 33, and in the 5–4 condition they were 33 and 26.

Necessarily, individuals perform at ceiling on their recall of the first red item in the list under this approach. Consequently, analysis of age group differences in recall focused only on the items following this start position. Serial position effects were not examined directly because, once the initial item is excluded, they are not particularly informative. In addition, because the number of individuals providing data in each condition varied (see above), condition was not included as a factor in this analysis initially. For each condition, the effect of age group on recall of the average performance on nonstart red items was significant: 7–2 condition, *F*(1, 91) = 7.57, *p* = .007, *MSE* = 0.07, 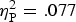; 6–3 condition, *F*(1, 81) = 14.11, *p* < .001, *MSE* = 0.08, 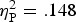; 5–4 condition, *F*(1, 57) = 14.41, *p* < .001, *MSE* = 0.09, 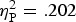. A further analysis then compared these averages across conditions for the 30 Year 1 and the 26 Year 3 children who provided data in all three conditions. In this analysis, the main effect of condition was significant, *F*(2, 108) = 53.34, *p* < .001, *MSE* = 0.03, 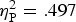, as was the main effect of age group, *F*(1, 54) = 11.50, *p* = .001, *MSE* = 0.15, 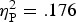. However, the interaction between these two factors was not significant, *F*(2, 108) = 1.84, *p* = .163, *MSE* = 0.03, 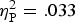. Post hoc comparisons showed that recall of the final red item in the 7–2 condition (*M* = .91, *SD* = .24) was significantly more accurate than the average of items 8 and 9 in the 6–3 condition (*M* = .83, *SD* = .27), which in turn was higher than average recall across the last three red items in the 5–4 condition (*M* = .58, *SD* = .33) [*t*(55) = 2.73, *p* = .009; *t*(55) = 7.25, *p* < .001, respectively].

### Discussion

In Experiment 1, we argued that there is a developmental improvement in children's ability to recall list-final items in free recall, but that this becomes manifest only when individuals began their recall with an item that was presented towards the end of the input list. Since participants were free to commence recall at any serial position, the number of trials providing data for this key analysis was not as high as it might have been and varied considerably across individuals. In Experiment 2, we specifically encouraged recall of late-presented items. One might argue that this reduces the task to a cued, rather than free, recall procedure, and that as a consequence the findings are not easily generalizable to other free-recall data. While the current task clearly does differ from the procedure employed in Experiment 1, it is important to note that nine items were again presented on each trial, and that these stimuli were all presented in the same way (using the same voice and with similar pictures) in both the blue and red phases of the trial. In addition, although many of the analyses above focus on just the recall of red items, it should not be forgotten that participants were instructed to try to remember both red and blue items on each trial.

Consequently, while the current procedure shares many similarities with recent free-recall studies that have presented adults with very short lists (Grenfell-Essam & Ward, 2012; Grenfell-Essam, Ward, & Tan, 2013; Ward et al., 2010), it does differ from that approach in important respects. In essence a contextual change prompts a particular focus on a subset of list-final items, and the recall instructions encourage the participant to begin their recall with these items, but other items are presented and are also probed. We would therefore argue that this paradigm does provide an appropriate means of assessing children's free-recall behaviour when they start their recall by outputting items from the end of the list. Indeed, the total number of items recalled by children in this experiment (red items in the red recall phase plus blue items in the blue recall phase) was broadly similar to that recalled from the standard free-recall task used in Experiment 1. Specifically, in Experiment 1, Year 1 and Year 3 children recalled 2.7 and 3.2 items on average from a 9-item list (see Experiment 1 results). Here the corresponding values were 2.1 and 3.0 items. Although the value for Year 1 children is somewhat lower in the current experiment, Year 3 children appear to be recalling a very similar number of items across the two tasks, suggesting that they do not assess fundamentally different processes.

Having said this, it is clear that children of different ages could differ either in their ability to understand the task instructions or in their ability to make use of the contextual cue that prompts a particular focus on these list-final items (cf. Davelaar, Goshen-Gottstein, Ashkenazi, Haarmann, & Usher, 2005). McCormack, Brown, Vousden, and Henson (2000) found a developmental improvement in the positional accuracy with which memoranda were recalled in children's immediate serial recall, and it is certainly possible that younger children might have a less distinct or accurate temporal representation of the start point of the red sublist. The fact that Year 1 children tended to recall somewhat fewer total items in this second experiment than they did in Experiment 1 might therefore suggest a failure to understand the task. However, this concern is mitigated considerably by the finding that the number of errors involving recall of a red list item in the blue recall phase (and vice versa) was extremely low and did not interact with age group. Consequently, it appeared that both age groups fully understood the task requirements and that even the younger children had a clear temporal representation of the start of the red sublist.

Consistent with this suggestion, and as Figure 6 shows, on a sizeable proportion of trials participants did commence recall of the probed (red) items with the first item in that set (cf. Figure 3). In fact, although this experiment included fewer Year 3 than Year 1 individuals overall, a comparable number of participants in each age group began their red phase recall with the first item in the red list on at least one trial in each condition (*n* = 30 and 26, respectively). This second experiment was therefore successful in increasing the likelihood of participants starting recall from a just-presented list at a certain position and therefore had even greater power than Experiment 1 to detect age differences in recall performance under these conditions. Furthermore, even if younger individuals did have a less precise temporal representation of this sublist starting position, the analyses that were restricted to trials on which recall did begin with the first item on the red list are particularly well placed to address this question. In this regard the results were clear, as Year 3 individuals were more successful at recalling red items than were Year 1 participants in each of the three conditions. If previous authors are correct in suggesting that just-presented items are held in primary memory in a free-recall task, particularly a task such as this in which participants are explicitly discouraged from selectively maintaining list-initial items for recall, then these findings clearly suggest that primary memory *does* develop in children.

We also found, through the analysis of +1 lag CRPs, that the two age groups had a comparable tendency to recall in forwards serial order. Although one must be wary of reading too much into a null effect, this implies that younger children were just as likely as older children to recall in forwards order, despite being less likely to start recall with the first item in the red sublist. We would argue that this pattern is consistent with the claim that younger children had a smaller recall *capacity* than older individuals. Put another way, older individuals were able to recall in forwards serial order a larger number of list-final items than were younger children and so tended to begin their recall nearer to the start of the red sublist. Indeed, the probability of first recall data plotted in Figure 6 show that Year 3 children tended to begin red phase recall with the first red item on the list when there were two or three red items on that list; in the 5–4 condition they were equally likely to begin recall with the first or the last red item. In contrast, Year 1 children showed a comparable tendency to start recall with either red item in the 7–2 condition, but were relatively unlikely to begin with the first red item when there were more than two red items in the list. This suggests that Year 1 children have a recall capacity that is not much more than two items, while Year 3 children tend to be able to recall at least three items from the list, values that are consistent with the estimates of total number of items recall across red and blue recall phases discussed above.^3^

^3^Given these estimates, one might question why Year 1 individuals did not show perfect recall in the 7–2 condition of the task (cf. Figure 7). However, Year 1 children's recall of the final list item on these trials, when their recall commenced with the 8th item on the list, was high, and any erroneous recall of this final item might be a reflection of the uncertainty about the type of list about to be presented on any trial. As the three different conditions were interleaved with one another, participants had no knowledge of the type of trial that was about to be presented. They might therefore have adopted an encoding strategy that maximized their chances of recalling any of the last four items on the list (rather than just the last two).


## GENERAL DISCUSSION

The main aim of these experiments was to examine age-related changes in children's ability to recall list-final items in free-recall tasks. This was done to test the claim that primary memory capacity is age invariant, which in turn rests on the assumption that list-final items in free recall are output first by virtue of being held in a form that makes them immediately available to conscious access. As outlined initially, the claim of developmental invariance in primary memory capacity runs counter to considerable evidence of developmental improvements on tests of immediate serial recall and measures of the size of children's focus of attention (e.g., Cowan et al., 2005), but is one that has been made both historically (e.g., Jablonski, 1974) and more recently (De Alwis et al., 2009) in the literature. It is also an important one to test, given the growing interest in the use of free recall as means of extracting primary memory capacity estimates for use in wider correlational studies (Gibson et al., 2009, 2013; Unsworth et al., 2011; Unsworth et al., 2010). The previous evidence for this position comes exclusively from the absence of age-related differences in children's recall of the final list items on an immediate free-recall task, a finding that we replicated in Experiment 1 (see Figure 1). However, even if one accepts that there are separate primary and secondary memory systems (and we discuss below the extent to which the current data do or do not support this view), the assumption that recall of list-final items are drawn from primary memory only holds if these items are output at the start of an individual's recall. Consequently, no strong inferences about primary memory capacity can be made from serial position curves that aggregate data across all trials regardless of output order. The present experiments therefore went considerably beyond any previous developmental work in this area by recording this crucial output order information and then using it to conditionalize serial position curves on the basis of different starting positions. In addition, Experiment 2 sought to prompt recall from a point near the end of the list in order to systematically examine age differences in free recall of a varying number of list-final items.

Both experiments provided clear evidence that children's ability to remember the later items on a list improved with age when these items were output at the start of an individual's recall sequence. In Experiment 1, when participants chose to begin recall with the 7th item on a 9-item list, younger individuals showed poorer recall of items 8 and 9 than did older children. The age group difference in recall of the 9th item on the list when participants started with the 8th item was not significant in Experiment 1. However, in Experiment 2, reliable age differences in final item recall were observed regardless of whether the probed (red) final sublist contained 2, 3, or 4 items. Furthermore, in this second experiment older children were more likely to begin their free recall with the first item within the red set of items than were younger children, providing converging evidence of their greater capacity for recall of these items. Again we note that the current data do not necessarily lead one to conclude that free recall must depend on the combination of distinguishable primary and secondary memory systems, and we return to this issue in more detail below. However, the key point to make here is that if one does hold this theoretical position, and therefore assumes that an individual's ability to recall list-final items at the start of their free-recall output depends on their primary memory capacity (Tulving & Colotla, 1970; Unsworth & Engle, 2007a), then the current data inevitably lead to the conclusion that primary memory capacity does develop with age.

A subsidiary aim of the current work was to reflect on whether the free-recall paradigm provides a reliable means of deriving estimates of individuals’ primary memory capacity, again assuming for the moment that such a system does underpin aspects of free-recall performance. In this respect, we believe the current experiments make two important contributions concerning the application of the Tulving and Colotla (1970) method to free-recall data. First, our evidence of a developmental change in capacity to recall from the end of a free-recall list implies that one should adopt a different criterion ITRI value for children of different ages if one is adopting this approach to the estimation of primary memory capacity. However, determining this value—which of course would itself be assumed to be directly related to primary memory capacity—is impossible without additional independent evidence of this capacity for any given age. This raises serious worries about circularity if such independent estimates are not available.

Second, the Tulving and Colotla (1970) approach assumes that participants begin their free recall with list-final items, and this assumption does not always hold. Whilst on long lists it may happen frequently (e.g., Ward et al., 2010), it is also not uncommon for participants to start recall with the first item on the just-presented list (Unsworth et al., 2011), and the greater this tendency the less meaningful will be any estimates of primary and secondary memory derived using the Tulving and Colotla approach. Crucially, in the first experiment reported here, older individuals were more likely to begin recall at the start of the list than were younger children (see Figure 3). Although we examined development over a relatively narrow age range and employed a relatively fast presentation rate to reduce the use of strategies, this greater likelihood of older individuals beginning recall at the start of the list could still potentially reflect the greater application of some form of strategy. This could be deeper encoding of initial list items, more use of rehearsal of initial list items to maintain them in immediate memory, or an awareness that beginning recall with otherwise hard to access items could result in superior performance. However, the point to emphasize is that any age-related difference in likelihood of starting free recall with the first item on the list, whatever its cause, would undermine the use of the Tulving and Colotla method in a developmental study. In turn, this means that methods of the form employed here, and which restrict analyses to trials on which recall does begin with list-final item, are needed if one wishes to extract potential indices of primary memory capacity from children's free recall.

It is therefore possible that future studies could extract estimates of primary memory capacity from children's immediate free-recall performance, provided that the previous two provisos are properly taken into consideration. The data from the current experiments would strongly suggest that clear age differences would be observed in these estimates of primary memory capacity. However, we also note that the current data do not count against a more unitary explanation of performance based on a developmental increase in other, more general factors. In particular, Experiment 2 provided some evidence that the total number of items that an individual could remember across both red and blue recall phases remained relatively constant across presentation conditions; somewhat lower totals were observed in the 7–2 condition, but total recall was comparable across the 6–3 and 5–4 conditions. One might argue that this simply reflects the capacity of primary memory, and that participants are holding different subsets of items (not always just the last few items) in primary memory on any given trial by choosing to selectively rehearse these items. However, a potentially different explanation of these data is that output interference is a major constraint on free-recall performance. There is considerable evidence that the effect of verbally outputting responses leads to forgetting of still-to-be-recalled items in both serial recall (see Tan & Ward, 2007) and free recall (Dalezman, 1976; Farrell, 2012; Roediger, 1974). In particular, Dalezman's (1976) study, on which the current Experiment 2 was loosely based, postcued adult participants to begin their free recall of a 15-item list with the first, middle, or last five items on the list. Dalezman found that while serial position curves were markedly affected by this manipulation, as would be expected and consistent with the current data, the overall number of items recalled across trials remained reasonably stable (see also Bruce & Papay, 1970; Katz, 1968; Unsworth et al., 2011). The clear implication of these data is that individuals’ ability to recall a certain number of items from a free-recall task is not necessarily moderated by the location of those items on the input list. In turn, this calls into question the suggestion that recall of list-final items depends on a separate memory system (i.e., primary memory) from that required for recall of earlier list items.

Having said this, Dalezman (1976) noted that the concepts of a limited-capacity store and of output interference are not mutually exclusive and argued that both are likely to constrain free-recall performance. A somewhat similar position was taken by Farrell (2012) in a recent computational model of both serial- and free-recall performance. Farrell rejected the need for a qualitative distinction between primary and secondary memory and instead suggested that an individual's episodic memory is temporally grouped into successive episodic clusters. Successful recall involves using contextual cues to access a given cluster; items in that cluster tend to then be recalled in forwards serial order. Output interference is also a key feature of the model. Under this analysis, recently presented (or indeed currently rehearsed) items are not held in an entirely distinct store. Instead, because the current cluster is associated with the current context at the point of recall, it does not need to be “accessed” before recall can be initiated. As a result, on long free-recall lists participants show a tendency to begin recall with list-final items and, provided the first item recalled is not the last item on the list, to then recall the remaining list items in forwards serial order. Although at heart a “unitary” account of free recall, this model does generate something akin to an output buffer that contains the currently open cluster of items. One might therefore draw a parallel between the size of this cluster and the capacity of primary memory under a dual-system model, and the currently accessible cluster would be assumed to be within any “region of direct attentional access” (Oberauer, 2002). Crucially, the size of this cluster can vary across participants, as can the degree of output interference experienced. Farrell's model has yet to be applied to developmental data, but in the context of the current experiments, the developmental differences observed here could be readily explained in terms of either age-related increases in cluster size (cf. Cowan et al., 2005; Cowan, Nugent, Elliot, & Saults, 2000) or decreases in susceptibility to output interference; both would lead to a greater total number of items being recalled from a trial among older than from a trial among younger children. The current results also clearly imply that age-related changes in the tendency to recall items from a cluster in forwards serial order is not likely to be the cause of developmental improvements in recall.

In summary, the present experiments have clearly demonstrated age-related changes in children's immediate free-recall performance. Crucially, these changes are not always limited to individuals’ ability to remember the initial items on the just-presented list, contrary to what has been suggested previously (Cole et al., 1971; De Alwis et al., 2009; Thurm & Glanzer, 1971). Rather, there is clear evidence that older children recall a greater total number of items than younger individuals from across the presented list, but without any noticeable increase in the tendency to recall in forwards serial order. In addition, older children are more likely to begin recall with initial list items than are younger children. This means that the greater recall capacity of older children is evident on these earlier list items, but is not apparent on later list items that are within the recall capacity of younger individuals, producing the previously reported age differences in the free-recall serial position curve when performance is aggregated across all trials. However, when all individuals begin their recall with list-final items, older children do recall more of these items than do younger children. Consequently, any model that distinguishes between primary and secondary memory to explain free-recall performance would be forced to conclude that primary memory does develop with age, contrary to previous claims in the free-recall literature. However, for the theoretical reasons outlined above, one is not obliged to assume that the ability to begin recall with list-final items depends on an entirely distinct primary memory system as opposed to more general factors such as output interference. Consequently further work is needed to link this aspect of children's free-recall performance to both specific measures of capacity and to more general measures such as children's ability to resist interference.

## References

[CIT0001] Atkinson R. C., Shiffrin R. M., Spence K. W., Spence J. T. (1968). Human memory: A proposed system and its control processes. *The psychology of learning and motivation*.

[CIT0002] Baddeley A. D. (1986). *Working memory*.

[CIT0003] Baddeley A. D., Hitch G., Bower G. H. (1974). Working memory. *The psychology of learning and motivation: Advances in research and theory*.

[CIT0004] Bayliss D. M., Jarrold C., Gunn D. M., Baddeley A. D. (2003). The complexities of complex span: Explaining individual differences in working memory in children and adults. *Journal of Experimental Psychology: General*.

[CIT0005] Bhatarah P., Ward G., Smith J., Hayes L. (2009). Examining the relationship between free recall and immediate serial recall: Similar patterns of rehearsal and similar effects of word length, presentation rate, and articulatory suppression. *Memory & Cognition*.

[CIT0006] Bjork R. A., Whitten W. B. (1974). Recency-sensitive retrieval processes in long-term free recall. *Cognitive Psychology*.

[CIT0007] Brown G. D. A., Neath I., Chater N. (2007). A temporal ratio model of memory. *Psychological Review*.

[CIT0008] Bruce D., Papay J. P. (1970). Primacy effect in single-trial free recall. *Journal of Verbal Learning and Verbal Behavior*.

[CIT0009] Cohen M. J. (1997). *Children's Memory Scale*.

[CIT0010] Cole M., Frankel F., Sharp D. (1971). Development of free recall learning in children. *Developmental Psychology*.

[CIT0011] Conway A. R. A., Kane M. J., Bunting M. F., Hambrick D. Z., Wilhelm O., Engle R. W. (2005). Working memory span tasks: A methodological review and user's guide. *Psychonomic Bulletin and Review*.

[CIT0012] Cowan N. (2001). The magical number 4 in short-term memory: A reconsideration of mental storage capacity. *Behavioral and Brain Sciences*.

[CIT0013] Cowan N., Elliot E. M., Saults J. S., Morey C., Mattox S., Hismjatullina A., Conway A. R.A. (2005). On the capacity of attention: Its estimation and its role in working memory and cognitive abilities. *Cognitive Psychology*.

[CIT0014] Cowan N., Nugent L. D., Elliot E. M., Saults J. S. (2000). Persistence of memory for ignored lists of digits: Areas of developmental constancy and change. *Journal of Experimental Child Psychology*.

[CIT0015] Cowan N., Saults J. S., Elliott E. M., Moreno M. V. (2002). Deconfounding serial recall. *Journal of Memory and Language*.

[CIT0016] Craik F. I., Birtwistle J. (1971). Proactive inhibition in free recall. *Journal of Experimental Psychology*.

[CIT0017] Crowder R. G., Morton J. (1969). Precategorical acoustic storage (PAS. *Perception & Psychophysics*.

[CIT0018] Cuvo A. J. (1975). Developmental differences in rehearsal and free recall. *Journal of Experimental Child Psychology*.

[CIT0019] Dalezman J. J. (1976). Effects of output order on immediate, delayed, and final recall performance. *Journal of Experimental Psychology: Human Learning and Memory*.

[CIT0020] Davelaar E. J., Goshen-Gottstein Y., Ashkenazi A., Haarmann H. J., Usher M. (2005). The demise of short-term memory revisited: empirical and computational investigations of recency effects. *Psychological Review*.

[CIT0021] De Alwis D., Myerson J., Hershey T., Hale S. (2009). Children's higher order cognitive abilities and the development of secondary memory. *Psychonomic Bulletin & Review*.

[CIT0022] Dempster F. N. (1981). Memory span: sources of individual and developmental differences. *Psychological Bulletin*.

[CIT0023] Dempster F. N., Rohwer W. D. (1983). Age-differences and modality effects in immediate and final free-recall. *Child Development*.

[CIT0024] De Smedt B., Janssen R., Bouwens K., Verschaffel L., Boets B., Ghesquière P. (2009). Working memory and individual differences in mathematics achievement: A longitudinal study from first grade to second grade. *Journal of Experimental Child Psychology*.

[CIT0025] Farrell S. (2010). Dissociating conditional recency in immediate and delayed free recall: A challenge for unitary models of recency. *Journal of Experimental Psychology: Learning, Memory, and Cognition*.

[CIT0026] Farrell S. (2012). Temporal clustering and sequencing in short-term memory and episodic memory. *Psychological Review*.

[CIT0027] Farrell S., Lewandowsky S. (2002). An endogenous distributed model of ordering in serial recall. *Psychonomic Bulletin & Review*.

[CIT0028] Gathercole S. E. (1999). Cognitive approaches to the development of short-term memory. *Trends in Cognitive Sciences*.

[CIT0029] Gibson B. S., Gondoli D. M., Flies A. C., Dobrzenski B. A., Unsworth N. (2009). Application of the dual-component model of working memory to ADHD. *Child Neuropsychology*.

[CIT0030] Gibson B. S., Gondoli D. M., Kronenberger W. G., Johnson A. C., Steeger C. M., Morrissey R. A. (2013). Exploration of an adaptive training regimen that can target the secondary memory component of working memory capacity. *Memory & Cognition*.

[CIT0031] Grenfell-Essam R., Ward G. (2012). Examining the relationship between free recall and immediate serial recall: The role of list length, strategy use, and test expectancy. *Journal of Memory and Language*.

[CIT0032] Grenfell-Essam R., Ward G., Tan L. (2013). The role of rehearsal on the output order of immediate free recall of short and long lists. *Journal of Experimental Psychology: Learning, Memory, and Cognition*.

[CIT0033] Hall D., Jarrold C., Towse J. N., Zarandi A. (2015). The developmental influence of primary memory on working memory and academic achievement. *Manuscript submitted for publication*.

[CIT0034] Hasher L., Clifton D. (1974). A developmental study of attribute encoding in free recall. *Journal of Experimental Child Psychology*.

[CIT0035] Howard M. W., Kahana M. J. (1999). Contextual variability and serial position effects in free recall. *Journal of Experimental Psychology: Learning, Memory, and Cognition*.

[CIT0036] Howard M. W., Kahana M. J. (2002). A distributed representation of temporal context. *Journal of Mathematical Psychology*.

[CIT0037] Jablonski E. M. (1974). Free recall in children. *Psychological Bulletin*.

[CIT0038] James W. (1890). *The principles of psychology*.

[CIT0039] Jonides J., Lewis R. L., Nee D. E., Lustig C. A., Berman M. G., Moore K. S. (2008). The mind and brain of short-term memory. *Annual Review of Psychology*.

[CIT0040] Kahana M. J. (1996). Associative retrieval processes in free recall. *Memory and Cognition*.

[CIT0041] Kane M. J., Hambrick D. Z., Conway A. R. A. (2005). Working memory capacity and fluid intelligence are strongly related constructs: comment on Ackerman, Beier, and Boyle (2005. *Psychological Bulletin*.

[CIT0042] Katz L. (1968). The limited-capacity hypothesis: Effects of sequence length and instructions in free recall. *Journal of Verbal Learning and Verbal Behavior*.

[CIT0043] Lehmann M., Hasselhorn M. (2007). Variable memory strategy use in children's adaptive intratask learning behavior: Developmental changes and working memory influences in free recall. *Child Development*.

[CIT0044] McCormack T., Brown G. D. A., Vousden J. I., Henson R. N. A. (2000). Children's serial recall errors: implications for theories of short-term memory development. *Journal of Experimental Child Psychology*.

[CIT0045] McElree B. (2006). Accessing recent events. *The Psychology of Learning and Motivation*.

[CIT0046] Moely B. E., Kail R. V., Hagen J. W. (1977). Organizational factors in the development of memory. *Perspectives on the development of memory and cognition*.

[CIT0047] Mogle J. A., Lovett B. J., Stawski R. S., Sliwinski M. J. (2008). What's so special about working memory?. An examination of the relationships among working memory, secondary memory, and fluid intelligence. *Psychological Science*.

[CIT0048] Morrison C. M., Chappell T. D., Ellis A. W. (1997). Age of acquisition norms for a large set of object names and their relation to adult estimates and other variables. *Quarterly Journal of Experimental Psychology Section A*.

[CIT0049] Murdock B. B. (1962). Direction of recall in short-term memory. *Journal of Verbal Learning and Verbal Behaviour*.

[CIT0050] Murdock B. B., Okada R. (1970). Interresponse times in single trial free recall. *Journal of Experimental Psychology*.

[CIT0051] Nee D. E., Jonides J. (2011). Dissociable contributions of prefrontal cortex and the hippocampus to short-term memory: Evidence for a 3-state model of memory. *Neuroimage*.

[CIT0052] Nevo E., Breznitz Z. (2011). Assessment of working memory components at 6 years of age as predictors of reading achievements a year later. *Journal of Experimental Child Psychology*.

[CIT0053] Oberauer K. (2002). Access to information in working memory: Exploring the focus of attention. *Journal of Experimental Psychology: Learning, Memory, and Cognition*.

[CIT0054] Oberauer K., Schulze R., Wilhelm O., Süß H.-M. (2005). Working memory and intelligence—their correlation and their relation: comment on Ackerman, Beier, and Boyle (2005. *Psychological Bulletin*.

[CIT0055] Öztekin I., Davachi L., McElree B. (2010). Are representations in working memory distinct from representations in long-term memory?: Neural evidence in support of a single store. *Psychological Science*.

[CIT0056] Page M. P. A., Norris D. (1998). The primacy model: a new model of immediate serial recall. *Psychological Review*.

[CIT0057] Roberts W. A. (1972). Free recall of word lists varying in length and rate of presentation: A test of total-time hypotheses. *Journal of Experimental Psychology*.

[CIT0058] Roediger H. (1974). Inhibiting effects of recall. *Memory & Cognition*.

[CIT0059] Shelton J. T., Elliott E. M., Matthews R. A., Hill B. D., Gouvier W. D. (2010). The relationships of working memory, secondary memory, and general fluid intelligence: Working memory is special. *Journal of Experimental Psychology: Learning, Memory, and Cognition*.

[CIT0060] Snodgrass J. G., Vanderwart M. (1980). A standardized set of 260 pictures: Norms for name agreement, image agreement, familiarity, and visual complexity. *Journal of Experimental Psychology: Human Learning and Memory*.

[CIT0061] Spurgeon J., Ward G., Matthews W. J. (2014). Examining the relationship between immediate serial recall and immediate free recall: Common effects of phonological loop variables but only limited evidence for the phonological loop. *Journal of Experimental Psychology: Learning, Memory, and Cognition*.

[CIT0062] Swanson H. L. (2011). Working memory, attention, and mathematical problem solving: A longitudinal study of elementary school children. *Journal of Educational Psychology*.

[CIT0063] Tan L., Ward G. (2007). Output order in immediate serial recall. *Memory and Cognition*.

[CIT0064] Thurm A. T., Glanzer M. (1971). Free recall in children: Long-term store vs short-term store. *Psychonomic Science*.

[CIT0065] Tulving E., Dixon T. R., Horton D. L. (1968). Theoretical issues in free recall. *Verbal behavior and general behavior theory*.

[CIT0066] Tulving E., Colotla V. A. (1970). Free recall of trilingual lists. *Cognitive Psychology*.

[CIT0067] Unsworth N., Brewer G., Spillers G. (2011). Inter- and intra-individual variation in immediate free recall: An examination of serial position functions and recall initiation strategies. *Memory*.

[CIT0068] Unsworth N., Engle R. W. (2006). Simple and complex memory spans and their relation to fluid abilities: Evidence from list-length effects. *Journal of Memory and Language*.

[CIT0069] Unsworth N., Engle R. W. (2007a). The nature of individual differences in working memory capacity: Active maintenance in primary memory and controlled search in secondary memory. *Psychological Review*.

[CIT0070] Unsworth N., Engle R. W. (2007b). On the division of short-term and working memory: An examination of simple and complex span and their relation to higher order abilities. *Psychological Bulletin*.

[CIT0071] Unsworth N., Spillers G. J., Brewer G. A. (2010). The contributions of primary and secondary memory to working memory capacity: An individual differences analysis of immediate free recall. *Journal of Experimental Psychology: Learning, Memory, and Cognition*.

[CIT0072] Ward G., Tan L., Grenfell-Essam R. (2010). Examining the relationship between free recall and immediate serial recall: The effects of list length and output order. *Journal of Experimental Psychology: Learning, Memory, and Cognition*.

[CIT0073] Waugh N. C., Norman D. A. (1965). Primary memory. *Psychological Review*.

